# Supplementary material for: Research on the improvement path of grassroots social governance innovation performance in China——Qualitative comparative analysis based on 35 cases
Source: PLoS One. 2024 Feb 21;19(2):e0297587. doi: 10.1371/journal.pone.0297587 (PMC10881008; doi:10.1371/journal.pone.0297587)
Supplement: Attachment — Submitted filename: Response to Reviewers.doc [file pone.0297587.s002.doc]

Dear Academic Editor Zhang,

We are very grateful for your consideration and suggestions about our manuscript entitled “How to achieve higher performance in grassroots social governance innovation in China？——fsQCA based on TOE framework” (Reference No: PONE-D-23-21194). We worked really hard on this article, which is related to whether a doctor can graduate on time. We have integrated the comments into the revised manuscript, and now we enclose the revised manuscript for your consideration for publication in PLOS ONE.

The followings are our responses with red fonts to the comments of the reviewers. After each the comments from the reviewers that are in italics, our answers are given. In addition, We updated the title logic, updated the cases and data, asked English professionals to polish the article, and highlighted it in color.

Best wishes,

Xiansheng Chen

**Academic Editor Zhang #:**

***1. Lines 26-41 are missing references.***

Answer: We agree with this point. We have added the following content and references in line 26-47 based on the comments of reviewer #1:

Grassroots social governance has attracted wide attention[1]. Grassroots society refers to the lowest and fundamental level of social organization and activities. It typically refers to the basic social units composed of residents, communities, villages, streets, etc., in urban or rural areas[2]. Grassroots society is characterized by direct engagement with residents, serving them, and addressing their practical issues[3].

1.	Raj G, Feola G, Hajer M, Runhaar H. Power and empowerment of grassroots innovations for sustainability transitions: A review. Environ Innov Soc Tr. 2022;43:375-92. http://doi.org/10.1016/j.eist.2022.04.009

2.	Lv L, Shi D. Innovative Development and Practice of Digital Rural Governance Model Based on Green Ecology. Sustainability-Basel. 2023;15(4). http://doi.org/10.3390/su15042955

3.	Yang Y, Wu F. The Sustainability of the Project-Driven Innovation of Grassroots Governance: Influencing Factors and Combination Paths. Sustainability-Basel. 2022;14(24). http://doi.org/10.3390/su142416862

4.	Li J, Zhan G, Dai X, Qi M, Liu B. Innovation and Optimization Logic of Grassroots Digital Governance in China under Digital Empowerment and Digital Sustainability. Sustainability-Basel. 2022;14(24). http://doi.org/10.3390/su142416470

5.	Farid M. Advocacy in Action: China's Grassroots NGOs as Catalysts for Policy Innovation. Stud Comp Int Dev. 2019;54(4):528-49. http://doi.org/10.1007/s12116-019-09292-3

6.	Qin Y. Grassroots governance and social development: theoretical and comparative legal aspects. Humanities & Social Sci Communications. 2023;10(1). http://doi.org/10.1057/s41599-023-01830-8

***2. Lines 91-99 are missing references.***

Answer: We agree. We updated the content on lines 96-104 and added the following references:

In the context of the TOE framework, information technology and platforms are considered important factors in enhancing innovation intensity[4]. Scientific and technological innovation can break down inherent institutional barriers between organizations and strengthen communication between participants through digital information systems or technology resource sharing platforms, thereby reducing information asymmetry and increasing the support capacity of information technology[30]. Organizational factors are key variables that affect technological innovation, and larger organizations tend to have advantages in resource allocation and innovation capabilities[31]. External pressure from government competitors and customers, as well as institutional cultural support, are also important factors influencing technological innovation[32].

4.	Li J, Zhan G, Dai X, Qi M, Liu B. Innovation and Optimization Logic of Grassroots Digital Governance in China under Digital Empowerment and Digital Sustainability. Sustainability-Basel. 2022;14(24). http://doi.org/10.3390/su142416470

30.	Shi Z, Wu YJ, Chiu YH, Chang TH. Research on the influence of technological innovation and technological application: Evidence from China. J Eng Technol Manage. 2022;63. http://doi.org/10.1016/j.jengtecman.2021.101670

31.	Sillig C. The role of ideology in grassroots innovation: An application of the arenas of development framework to organic in Europe. Ecol Econ. 2022;191. http://doi.org/10.1016/j.ecolecon.2021.107252

32.	Mamonov S, Peterson R. The role of IT in organizational innovation-A systematic literature review. J Strategic Inf Syst. 2021;30(4). http://doi.org/10.1016/j.jsis.2021.101696

***3. The discussion section is missing references. And the conclusion should be discussed below.***

Answer: Thanks for your suggestion, we have adjusted the position of the conclusion and discussion section, enriched the discussion content and updated the references：

Based on the above research conclusions, this paper provides the following management implications for government departments to improve the innovation performance of grassroots social governance:

First, the openness of the internal environment is an indispensable key factor to improve the innovation performance of grassroots social governance. In the information age, the openness of the internal environment of the government requires it to hold a more open and inclusive attitude towards new things including information technology, and be able to flexibly perceive, actively respond to and accept the opportunities and challenges that information technology may bring[44]. It reflects the factors of organizational system culture. From the historical development experience, politics and economy are closely intertwined, whether it is Milton Friedman's promotion of economic freedom to political freedom [45], The mutual support between the openness of power in the economic field and the openness of power in the political field by Douglass C. North both demonstrated a high positive correlation between the openness of environment within the government and the degree of marketization[46]. Therefore, the government should follow the development of the market economy and further improve the degree of marketization.

Second, the existence of concurrent synergies among technology, organization and environment reveals the complexity of improving innovation performance of grassroots social governance. Although technology, organization and environment are all important factors affecting innovation performance, the focus on a single factor is difficult to achieve the expected effect, and the adaptation of multiple factors should be paid attention to from an "overall perspective" and corresponding measures should be adopted "according to local conditions"[47]. We will develop differentiated paths for community-level social governance innovation. The government can make efforts from three aspects: First, strengthen the technical support for grassroots social governance innovation[48]. We will apply advanced technologies such as big data, blockchain, Internet of Things, and 5G to multiple scenes of grassroots social governance, and strive for overtaking on corners and leapfrog development. The second is to strengthen the organizational construction of grassroots social governance innovation[49]. In the public sector, especially in government organizations, the willingness of lower-level governments to innovate and develop depends to a certain extent on the financial resources and policy support provided by higher-level governments, which have a strong dependence on higher-level government organizations. Thus, top-down political pressure is the most dominant external driver of applied innovation in the public sector[50]. China's vertical governments at all levels are in essence a "relationship between superiors and subordinates". The policy support of higher governments to lower governments reflects a certain attribute of political pressure, which can bring various resources and pressures to lower governments. Therefore, higher governments should actively encourage and support lower governments to promote innovative practice of grassroots social governance. In addition, studies have shown that local governments' self-importance has a significant positive impact on innovation performance. Local governments should attach importance to grassroots social governance innovation practices and formulate development strategies, norms and measures according to local conditions[51]. The third is to optimize the external environment for grassroots social governance innovation, improve its own competitiveness, actively respond to public demand, build an institutional environment with multi-subject participation, and create a grassroots social governance community with co-construction of subjects, co-governance of resources and shared benefits[52].

Third, whether from the perspective of the whole country or from the perspective of regional differences, there is more than one "satisfactory solution" for the improvement of grassroots social governance innovation performance or grassroots social governance innovation, that is to say, there is no "one-size-fits-all" generation path, which can be started from expanding the openness of the internal environment[52]. It can also promote innovation in social governance at the grassroots level from the aspects of technological innovation capacity, technical facility support, promotion by the higher level government, and their own importance. There are multiple paths to improve innovation performance, not a single one[54]. Therefore, local governments should select appropriate paths and targeted measures based on local development scenarios and governance resources, and find an efficient way to innovate grassroots social governance in China.

This paper has the following shortcomings and provides directions for future research: firstly, this paper only considers preconditions such as technology, organisation and environment, but there are many factors that affect innovation performance. Future research can incorporate factors such as strategic orientation and organisational resources to study the influencing factors of innovation performance more comprehensively. Second, 35 grassroots social governance innovation cases were selected for this paper, and the generalisability of the results of the qualitative comparative analysis in terms of application is limited by the sample size. In the future, more data can be collected for further analysis.

44.	Pang SL, Dou ST, Li H. Synergy effect of science and technology policies on innovation: Evidence from China. Plos One. 2020;15(10). http://doi.org/10.1371/journal.pone.0240515

45.	Turner R. Un/inhibited organism: Political power, psychophysiology, and techniques.; 2009.

46.	Wang W, Wen J, Luo ZG, Luo WY. How does environmental punishment affect regional green technology innovation?-Evidence from Chinese Provinces. Plos One. 2023;18(7). http://doi.org/10.1371/journal.pone.0288080

47.	Liang LW, Wang ZB, Luo D, Wei Y, Sun JW. Synergy effects and it's influencing factors of China's high technological innovation and regional economy. Plos One. 2020;15(5). http://doi.org/10.1371/journal.pone.0231335

48.	Yang HC, Li LS, Liu YB. The effect of manufacturing intelligence on green innovation performance in China. Technol Forecast Soc. 2022;178. http://doi.org/10.1016/j.techfore.2022.121569

49.	Rehman SU, Ashfaq K, Bresciani S, Giacosa E, Mueller J. Nexus among intellectual capital, interorganizational learning, industrial Internet of things technology and innovation performance: a resource-based perspective. J Intellect Cap. 2023;24(2):509-34. http://doi.org/10.1108/JIC-03-2021-0095

50.	Andersen SC, Jakobsen ML. Political Pressure, Conformity Pressure, and Performance Information as Drivers of Public Sector Innovation Adoption. Int Public Manag J. 2018;21(2):213-42. http://doi.org/10.1080/10967494.2018.1425227

51.	He C, Wang YP, Tang K. Impact of Low-Carbon City Construction Policy on Green Innovation Performance in China. Emerg Mark Financ Tr. 2023;59(1):15-26. http://doi.org/10.1080/1540496X.2022.2089019

52.	Xu JH, He SY. Can Grid Governance Fix the Party-state's Broken Windows? A Study of Stability Maintenance in Grassroots China. China Quart. 2022;251:843-65. http://doi.org/10.1017/S0305741022000509

53.	Bianchi I. Empowering policies for grassroots welfare initiatives: Blending social innovation and commons theory. Eur Urban Reg Stud. 2023;30(2):107-20. http://doi.org/10.1177/09697764221129532

54.	Qian X, Cai Y, Yin C. Driving Force of Grassroots Self-governance in Beijing's Neighborhoods: Social Capital, Community Network and Community Service Motivation. Lex Localis. 2019;17(1):159-77. http://doi.org/10.4335/17.1.159-177(2019)

***4. There are too few and old references.***

Answer: Thanks for your suggestion, we have updated the content and references

**Response to Reviewer #1:**

***In China, grassroots social governance innovation is a very fashionable research topic, but many studies are vague about the concept of grassroots society or grassroots, and such studies lack authenticity. The article does not clearly define and discuss the concept of grassroots society. Suggest the author to add.***

Answer: We agree with this point. In lines 27-31, we have added an explanation about grassroots society:

“Grassroots social governance has attracted wide attention. Grassroots society refers to the lowest and fundamental level of social organization and activities. It typically refers to the basic social units composed of residents, communities, villages, streets, etc., in urban or rural areas. Grassroots society is characterized by direct engagement with residents, serving them, and addressing their practical issues.”

1.	Raj G, Feola G, Hajer M, Runhaar H. Power and empowerment of grassroots innovations for sustainability transitions: A review. Environ Innov Soc Tr. 2022;43:375-92. http://doi.org/10.1016/j.eist.2022.04.009

2.	Lv L, Shi D. Innovative Development and Practice of Digital Rural Governance Model Based on Green Ecology. Sustainability-Basel. 2023;15(4). http://doi.org/10.3390/su15042955

3.	Yang Y, Wu F. The Sustainability of the Project-Driven Innovation of Grassroots Governance: Influencing Factors and Combination Paths. Sustainability-Basel. 2022;14(24). http://doi.org/10.3390/su142416862

4.	Li J, Zhan G, Dai X, Qi M, Liu B. Innovation and Optimization Logic of Grassroots Digital Governance in China under Digital Empowerment and Digital Sustainability. Sustainability-Basel. 2022;14(24). http://doi.org/10.3390/su142416470

5.	Farid M. Advocacy in Action: China's Grassroots NGOs as Catalysts for Policy Innovation. Stud Comp Int Dev. 2019;54(4):528-49. http://doi.org/10.1007/s12116-019-09292-3

6.	Qin Y. Grassroots governance and social development: theoretical and comparative legal aspects. Humanities & Social Sci Communications. 2023;10(1). http://doi.org/10.1057/s41599-023-01830-8

**Response to Reviewer #2:**

***At present, social governance innovation is an important issue. Although this study has achieved some results, however, some problems still remain. I put forward some suggestions for the author's reference.***

***1.Theory and analysis framework: This part should be further improved. In particular, the development and current status of the theory, as well as the elaboration of current related research.***

Answer: We agree, In the theoretical part, we added the following contents and references, and systematically expounded the research status：

TOE theoretical framework (Technology-organization-Environment) was developed by Tornatzky and Fleischer in 1990 and was originally used to analyze the factors affecting the adoption of innovative technologies by enterprises. It is essentially a comprehensive analysis framework based on the Technology application context[24].

In the context of the TOE framework, information technology and platforms are considered important factors in enhancing innovation intensity[4]. Scientific and technological innovation can break down inherent institutional barriers between organizations and strengthen communication between participants through digital information systems or technology resource sharing platforms, thereby reducing information asymmetry and increasing the support capacity of information technology[30]. Organizational factors are key variables that affect technological innovation, and larger organizations tend to have advantages in resource allocation and innovation capabilities[31]. External pressure from government competitors and customers, as well as institutional cultural support, are also important factors influencing technological innovation[32].

4.	Li J, Zhan G, Dai X, Qi M, Liu B. Innovation and Optimization Logic of Grassroots Digital Governance in China under Digital Empowerment and Digital Sustainability. Sustainability-Basel. 2022;14(24). http://doi.org/10.3390/su142416470

24.	Tornatzky LG, Fleischer M, Chakrabarti AK. Processes of technological innovation.: Lexington books; 1990.

30.	Shi Z, Wu YJ, Chiu YH, Chang TH. Research on the influence of technological innovation and technological application: Evidence from China. J Eng Technol Manage. 2022;63. http://doi.org/10.1016/j.jengtecman.2021.101670

31.	Sillig C. The role of ideology in grassroots innovation: An application of the arenas of development framework to organic in Europe. Ecol Econ. 2022;191. http://doi.org/10.1016/j.ecolecon.2021.107252

32.	Mamonov S, Peterson R. The role of IT in organizational innovation-A systematic literature review. J Strategic Inf Syst. 2021;30(4). http://doi.org/10.1016/j.jsis.2021.101696

***2.The format of 320-334、355-359 and line376-382 should be adjusted.***

Answer: Thank you for your suggestion, We readjusted the paragraphs and spacing and content.

***3. The Discussion should be further enriched. The current Discussion is not enlightening. In addition to the discussion of the results, there should be an extension of the author's thinking to enlighten the reader. This is one of the most important things in the manuscript.***

Answer: We agree. We have enriched the discussion section and added references. The specific changes and references are as follows:

5.1 Discussion

Based on the above research conclusions, this paper provides the following management implications for government departments to improve the innovation performance of grassroots social governance:

First, the openness of the internal environment is an indispensable key factor to improve the innovation performance of grassroots social governance. In the information age, the openness of the internal environment of the government requires it to hold a more open and inclusive attitude towards new things including information technology, and be able to flexibly perceive, actively respond to and accept the opportunities and challenges that information technology may bring[44]. It reflects the factors of organizational system culture. From the historical development experience, politics and economy are closely intertwined, whether it is Milton Friedman's promotion of economic freedom to political freedom [45], The mutual support between the openness of power in the economic field and the openness of power in the political field by Douglass C. North both demonstrated a high positive correlation between the openness of environment within the government and the degree of marketization[46]. Therefore, the government should follow the development of the market economy and further improve the degree of marketization.

Second, the existence of concurrent synergies among technology, organization and environment reveals the complexity of improving innovation performance of grassroots social governance. Although technology, organization and environment are all important factors affecting innovation performance, the focus on a single factor is difficult to achieve the expected effect, and the adaptation of multiple factors should be paid attention to from an "overall perspective" and corresponding measures should be adopted "according to local conditions"[47]. We will develop differentiated paths for community-level social governance innovation. The government can make efforts from three aspects: First, strengthen the technical support for grassroots social governance innovation[48]. We will apply advanced technologies such as big data, blockchain, Internet of Things, and 5G to multiple scenes of grassroots social governance, and strive for overtaking on corners and leapfrog development. The second is to strengthen the organizational construction of grassroots social governance innovation[49]. In the public sector, especially in government organizations, the willingness of lower-level governments to innovate and develop depends to a certain extent on the financial resources and policy support provided by higher-level governments, which have a strong dependence on higher-level government organizations. Thus, top-down political pressure is the most dominant external driver of applied innovation in the public sector[50]. China's vertical governments at all levels are in essence a "relationship between superiors and subordinates". The policy support of higher governments to lower governments reflects a certain attribute of political pressure, which can bring various resources and pressures to lower governments. Therefore, higher governments should actively encourage and support lower governments to promote innovative practice of grassroots social governance. In addition, studies have shown that local governments' self-importance has a significant positive impact on innovation performance. Local governments should attach importance to grassroots social governance innovation practices and formulate development strategies, norms and measures according to local conditions[51]. The third is to optimize the external environment for grassroots social governance innovation, improve its own competitiveness, actively respond to public demand, build an institutional environment with multi-subject participation, and create a grassroots social governance community with co-construction of subjects, co-governance of resources and shared benefits[52].

Third, whether from the perspective of the whole country or from the perspective of regional differences, there is more than one "satisfactory solution" for the improvement of grassroots social governance innovation performance or grassroots social governance innovation, that is to say, there is no "one-size-fits-all" generation path, which can be started from expanding the openness of the internal environment[52]. It can also promote innovation in social governance at the grassroots level from the aspects of technological innovation capacity, technical facility support, promotion by the higher level government, and their own importance. There are multiple paths to improve innovation performance, not a single one[54]. Therefore, local governments should select appropriate paths and targeted measures based on local development scenarios and governance resources, and find an efficient way to innovate grassroots social governance in China.

This paper has the following shortcomings and provides directions for future research: firstly, this paper only considers preconditions such as technology, organisation and environment, but there are many factors that affect innovation performance. Future research can incorporate factors such as strategic orientation and organisational resources to study the influencing factors of innovation performance more comprehensively. Second, 35 grassroots social governance innovation cases were selected for this paper, and the generalisability of the results of the qualitative comparative analysis in terms of application is limited by the sample size. In the future, more data can be collected for further analysis.

44.	Pang SL, Dou ST, Li H. Synergy effect of science and technology policies on innovation: Evidence from China. Plos One. 2020;15(10). http://doi.org/10.1371/journal.pone.0240515

45.	Turner R. Un/inhibited organism: Political power, psychophysiology, and techniques.; 2009.

46.	Wang W, Wen J, Luo ZG, Luo WY. How does environmental punishment affect regional green technology innovation?-Evidence from Chinese Provinces. Plos One. 2023;18(7). http://doi.org/10.1371/journal.pone.0288080

47.	Liang LW, Wang ZB, Luo D, Wei Y, Sun JW. Synergy effects and it's influencing factors of China's high technological innovation and regional economy. Plos One. 2020;15(5). http://doi.org/10.1371/journal.pone.0231335

48.	Yang HC, Li LS, Liu YB. The effect of manufacturing intelligence on green innovation performance in China. Technol Forecast Soc. 2022;178. http://doi.org/10.1016/j.techfore.2022.121569

49.	Rehman SU, Ashfaq K, Bresciani S, Giacosa E, Mueller J. Nexus among intellectual capital, interorganizational learning, industrial Internet of things technology and innovation performance: a resource-based perspective. J Intellect Cap. 2023;24(2):509-34. http://doi.org/10.1108/JIC-03-2021-0095

50.	Andersen SC, Jakobsen ML. Political Pressure, Conformity Pressure, and Performance Information as Drivers of Public Sector Innovation Adoption. Int Public Manag J. 2018;21(2):213-42. http://doi.org/10.1080/10967494.2018.1425227

51.	He C, Wang YP, Tang K. Impact of Low-Carbon City Construction Policy on Green Innovation Performance in China. Emerg Mark Financ Tr. 2023;59(1):15-26. http://doi.org/10.1080/1540496X.2022.2089019

52.	Xu JH, He SY. Can Grid Governance Fix the Party-state's Broken Windows? A Study of Stability Maintenance in Grassroots China. China Quart. 2022;251:843-65. http://doi.org/10.1017/S0305741022000509

53.	Bianchi I. Empowering policies for grassroots welfare initiatives: Blending social innovation and commons theory. Eur Urban Reg Stud. 2023;30(2):107-20. http://doi.org/10.1177/09697764221129532

54.	Qian X, Cai Y, Yin C. Driving Force of Grassroots Self-governance in Beijing's Neighborhoods: Social Capital, Community Network and Community Service Motivation. Lex Localis. 2019;17(1):159-77. http://doi.org/10.4335/17.1.159-177(2019)

***4. Increased emphasis on research contributions/values, limitations and future research directions.***

Answer: Thank you for your valuable advice, We have enriched the research contributions/values, limitations and future research directions. The specific changes and references are as follows:

This paper has the following shortcomings and provides directions for future research: firstly, this paper only considers preconditions such as technology, organisation and environment, but there are many factors that affect innovation performance. Future research can incorporate factors such as strategic orientation and organisational resources to study the influencing factors of innovation performance more comprehensively. Second, 35 grassroots social governance innovation cases were selected for this paper, and the generalisability of the results of the qualitative comparative analysis in terms of application is limited by the sample size. In the future, more data can be collected for further analysis. Therefore, this study has important theoretical and practical value, and can provide new ideas and methods for the research of management and social governance.

***5. References must be further increased, especially those within the last three years.***

Answer: Thanks for your suggestion, we have updated the content and references.
