# Supplementary material for: Research on the improvement path of grassroots social governance innovation performance in China——Qualitative comparative analysis based on 35 cases
Source: PLoS One. 2024 Feb 21;19(2):e0297587. doi: 10.1371/journal.pone.0297587 (PMC10881008; doi:10.1371/journal.pone.0297587)
Supplement: Attachment — Submitted filename: Response to Reviewers.doc [file pone.0297587.s003.doc]

Dear Editor,

I hope this message finds you well. I am writing to provide an update regarding the manuscript with the reference number PONE-D-23-21194R1, titled "Research on the Improvement Path of Grassroots Social Governance Innovation Performance in China - Qualitative comparative analysis based on 35 cases," which was submitted to PLOS ONE.

This article plays a key role in whether I can successfully graduate on time. I would like to express my gratitude for the careful consideration given to our manuscript. We have thoroughly reviewed the comments raised during the review process and have made significant revisions to address them.

I would like to bring to your attention that we recently received comments from Reviewer #3, which were not previously communicated to us. We apologize for any confusion caused by this oversight. However, I want to assure you that we have taken these comments into account and have made additional modifications to further strengthen the manuscript.

Thank you for your understanding and consideration. We are very fortunate if the revised manuscript now meets PLOS ONE's publication criteria. We look forward to receiving further guidance and instructions for the submission of the revised version.

Best wishes,

Nana Song，Longshun Xu，Xiansheng Chen，Huange Xu, Shuoliang Jiang

**In response to Reviewer #3's concerns, we have made the following improvements:**

***1. The language of the paper needs to be more concise.***

Answer: Thanks for your suggestion. We asked our English major friends to help us polish the whole article. Specific changes have been marked.

***2.Regarding the data and methods, what is the data source? These aspects need more detailed explanation. There could also be graphical preseantations of the trend of variables.***

Answer: We agree. An additional explanation is given in the paper：

“According to the above case selection criteria, this paper selects 35 grassroots social governance innovation cases from the "Top Ten Social Governance Innovation in China" award selected by the China All-round Well-off Forum and official news reports of governments at all levels. The data based on the values of each variable come from official data, journal data, and Internet media such as Xinhuanet, NetEase, Sohu and Ifeng.com”.

In addition, due to the word limit, the 35 cases selected were not shown in the paper, and the data provided clearly showed the source of the cases, as well as variable assignment and coding. Specific cases are as follows:

Table1: study case information

| **caseid** | **Case** |
| --- | --- |
| case1 | Tong 'an District, Xiamen: Building a miniature "closed system" for community governance |
| case2 | Rizhao City, Shandong Province: Build a four-level digital platform |
| case3 | Wuchang District, Wuhan City, Hubei Province: "three-community cooperation" promotes diverse community governance |
| case4 | Yangpu District, Shanghai: "Community Center" |
| case5 | Huaian City, Jiangsu Province, Letters and Calls Bureau: "126" new mode of letters and calls work |
| case6 | Xiangyang City, Hubei Province: public recommendation and competitive selection of county Party secretaries and county (district) chiefs |
| case7 | Liupanshui City, Guizhou Province: The "Three iron measures" of political and legal organs serve the non-public economy to promote social harmony |
| case8 | Zhejiang Provincial Civil Affairs Department: "Village rules promote harmony, social conventions ensure governance" |
| case9 | The Organization Department of the CPC Shaoxing Municipal Committee: Party building leads the rural revitalization "five-star standard 3A striving" working mechanism |
| case10 | Ningjiang District, Songyuan City, Jilin Province: Tuanjie Street strives to improve the community safety prevention system |
| case11 | Harbin City, Heilongjiang Province: Community neighborly center practice innovation |
| case12 | Deqing County, Zhejiang Province: urban and rural domestic waste treatment integration project |
| case13 | Hangzhou, Zhejiang Province: Urban brain "Hangzhou Solution" |
| case14 | Liyang City, Jiangsu Province: "people's hall" set up in every village |
| case15 | Tianya District, Sanya City, Hainan Province: "Joint Command Center" |
| case16 | Shenzhen Pingshan New District: the application of big data in social governance |
| case17 | Xigang District of Dalian City: 365 work system |
| case18 | Dongguan, Guangdong Province: Innovation and promotion of points-based management constantly improve the grass-roots governance system |
| case19 | Jinan City, Shandong Province: Establish the "four social linkage" mechanism |
| case20 | Nanjing, Jiangsu Province: Party Building Alliance of elderly care Service organizations |
| case21 | Yinchuan City, Ningxia: Party building leads grassroots governance |
| case22 | Inner Mongolia Autonomous Region: Building Safe Chifeng |
| case23 | Chengdu, Sichuan Province: Tianfu citizen Cloud "Top ten" citizen reputation series service projects |
| case24 | Shaoxing City, Zhejiang Province: remote convenience service center |
| case25 | Wuxi City, Jiangsu Province: The first river chief system in China |
| case26 | Wulong District of Chongqing: Optimizing grid governance to promote the construction of social governance system |
| case27 | Jiuquan City, Gansu Province: implement the working mode of "one office, four rooms and one center" |
| case28 | Donglan County, Hechi City, Guangxi Autonomous Region: Seven people, one station and one position working method |
| case29 | Guizhou Sandu Zhonghe: Explore the "1+2" work method to build the "Five Bridges" to build the "five homes" |
| case30 | Hefei, Anhui Province: Rule of law thinking improves people's livelihood |
| case31 | Yan 'an City, Shaanxi Province: implement the "problem wall + echo wall" system |
| case32 | Jinzhong City, Shanxi Province: "Five checks and five treatments" to improve the sense of gain in poverty alleviation |
| case33 | Songgang Street, Baoan District, Shenzhen City, Guangdong Province: "One grid multiple" grid grassroots governance |
| case34 | Jiangbei District, Ningbo City, Zhejiang Province: "One thing" integrated reform stimulates new momentum of grassroots governance |
| case35 | Hunchun City, Yanbian Prefecture, Jilin Province: Build a team of professional community workers |

The specific research method is further clearly pointed out in the paper, the qualitative comparative analysis method is used in this paper, and the advantages of the method, why the method is used, and the technical principle of the method are explained. The specific contents are as follows:

“In this paper, we abandon the traditional binary linear model and try to use fuzzy set qualitative comparative analysis (fsQCA) from the perspective of configuration to analyze the multiple driving path to improve the innovation performance of grassroots social governance. The qualitative comparative analysis method can solve the data analysis of 10-60 small and medium-sized samples, and find out the logical relationship between the innovation performance of grassroots social governance under different matching modes of ante-cause conditions. The fsQCA method has the following considerations”

***3.The theoretical basis for the mechanisms presented in this study is very weak and unconvincing. The novelty of this manuscript is very inadequate. There are many similar studies.***

Answer: Thanks for your suggestion. I have modified and supplemented the theoretical basis. Details are as follows:

“The academic circles mainly study the innovation of grassroots social governance from three perspectives。The first is to study the barriers to innovation in grassroots social governance from the perspective of problem interpretation.。From the government level, the government is the leader of grassroots social governance innovation, and grassroots social governance innovation has a strong political nature, and the government pays more attention to efficiency but ignores democracy[5].It makes the grassroots social governance innovation lack vitality. At the same time, the dislocation and absence of functions of grass-roots governments, insufficient supply of resources, and lagging management reduce the innovation performance to some extent[6].From the perspective of the public, grassroots social governance innovation is easy to be covered up by technocracy and exclusivity, and the public is rarely the core of previous deliberation, decision-making and development[7], and there is no institutional guarantee for public participation in grassroots social governance innovation; From the perspective of innovation itself, grassroots social governance innovation is not sustainable, innovation subjects lack enthusiasm, innovation projects lack operability, and the use of innovative technologies is insufficient. The second is to study the influencing factors of grassroots social governance innovation from the perspective of causality. Based on the political dimension, some scholars have discussed the impact of government authority, political support[8], organizational structure, power structure, legal structure, action structure, grassroots social governance system, and the "trinity" model on grassroots social governance innovation. Other scholars have studied the interactions between personal attributes, political leaders and their environment[9], top-down political mandates and policy pressures, civil rights and civic participation[10], and the overlap of innovative activities with existing social structures and social needs[11]. The net effect of other factors on grassroots social governance innovation. The third is to study the innovation path of grassroots social governance based on the goal-oriented perspective. From the government level, the government should optimize the grid management system in governance innovation, build the network structure of actors, and form innovation synergy[12].The government should further integrate various resources, respect the interests of multiple subjects, strengthen political commitment, optimize the collaborative governance model, improve the grassroots governance innovation system, and form a grassroots governance innovation community. From the public level, improve the enthusiasm of the public to participate in grassroots social governance innovation, improve the "government-society" cooperation mechanism, strengthen technological innovation and application, and stimulate the vitality of grassroots social governance innovation[13];From the perspective of the innovation process, the innovation process of grassroots social governance itself is conducive to fostering a more general democracy, which requires strengthening the use of technology, coordinating the interests of all parties, and improving infrastructure[14].The innovation process needs to be more open to public scrutiny and wider participation.”

We added the following literature:

“5. Stirling A. Towards Innovation Democracy? Participation, Responsibility and Precaution in Innovation Governance. Participation, Responsibility and Precaution in Innovation Governance.(November 2014). SWPS. 2014;24.

6.	Li A. Analysis of the Problems and Countermeasures of Grass-Roots Government in Community Governance. Academic Journal of Management and Social Sciences. 2023;5(1):131-7.

7.	Kearnes M, Chilvers J. Remaking Participation in Science and Democracy. Science, Technology, and Human Values. 2020;45(3).

8.	Cinar E, Trott P, Simms C. A systematic review of barriers to public sector innovation process. Public Manag Rev. 2019;21(2):264-90.

9.	Wynen J, Verhoest K, Ongaro E, Van Thiel S, In CWTC. Innovation-oriented culture in the public sector: Do managerial autonomy and result control lead to innovation? Public Manag Rev. 2014;16(1):45-66.

10.	Crosby BC, T Hart P, Torfing J. Public value creation through collaborative innovation. Public Manag Rev. 2017;19(5):655-69.

11.	Korac S, Saliterer I, Walker RM. Analysing the environmental antecedents of innovation adoption among politicians and public managers. Public Manag Rev. 2017;19(4):566-87.

12.	Mittelstaedt JC. The grid management system in contemporary China: Grass-roots governance in social surveillance and service provision. China Inform. 2022;36(1):3-22.

13.	Huang X, Zhou L. “Paired competition”: A new mechanism for the innovation of urban grassroots governance. Chinese Journal of Sociology. 2023;9(1):3-45.

14.	Smith A, Stirling A. Innovation, sustainability and democracy: An analysis of grassroots contributions. Journal of Self-Governance and Management Economics. 2018;6(1):64-97.”

***4.In addition, the research purpose and research framework of the manuscript are unclear. There are unaddressed issues in the paper that bear on the central conclusions of the paper.***

Answer: Thank you for your valuable advice. We re-sorted out the research framework, and according to the research framework to conduct problem research, and finally analyze and solve the problem. In the introduction of this paper, based on the practice status of grassroots social governance innovation in China and the research status of grassroots social governance innovation theory, two research objectives of this paper are proposed. In addition, the purpose and framework of the research are further explained in the theoretical and analytical framework. Details are as follows:

“The first is to study the barriers to innovation in grassroots social governance from the perspective of problem interpretation.” “The second is to study the influencing factors of grassroots social governance innovation from the perspective of causality. Based on the political dimension, some scholars have discussed the impact of government authority, political support, organizational structure, power structure, legal structure, action structure, grassroots social governance system, and the "trinity" model on grassroots social governance innovation.”、“The third is to study the innovation path of grassroots social governance based on the goal-oriented perspective. From the government level, the government should optimize the grid management system in governance innovation, build the network structure of actors, and form innovation synergy”

*Line 91-97* “Expanding existing studies from the three dimensions of technical factors, organizational factors and environmental factors, focusing on the single factor influence of grassroots social governance innovation performance, ignoring the lack of research on the combination of multiple factors, and then revealing the core influencing factors of grassroots social governance innovation to achieve high performance and the substitution effect between different paths. It provides reference for local governments to adopt differentiated innovation paths of grassroots social governance according to local conditions.”

The specific framework is as follows:

**“2.1 Technical factors and grassroots social governance innovation performance**

In order to achieve higher performance in grassroots social governance innovation, it is necessary to break through the problem of insufficient use of technology in the traditional governance model, which requires increasing technological innovation efforts and improving technological innovation ability. Especially with the rapid development of network information technology, governance technology has become an important tool to promote grassroots social governance innovation, and digital technology has become a key driving force to improve grassroots social governance innovation performance[20]. Based on the actual situation of China's grass-roots social governance, China's grass-roots population is large, grass-roots problems are complex, and it is more necessary to promote technological innovation and increase the application of technology, so as to improve the innovation performance of grass-roots social governance. Most scholars view input-output, cost-benefit and other perspectives[21]. Focus on whether technology application can reduce governance costs and whether technology empowerment can promote performance. The technology application of grassroots social governance needs to rely on the support of technological innovation ability and technical facilities. Generally speaking, technological innovation capability is the core competitiveness of regional economic development and grassroots governance, and it represents the breadth and depth of grassroots social governance innovation, while technological facility platform is an important form of technology application, and an important tool to facilitate grassroots social governance and improve grassroots social governance performance[22].

**2.2 Organizational factors and grassroots social governance innovation performance**

Grassroots social governance innovation behavior is the specific implementation of strategic decision-making, and grassroots social governance innovation strategic decision-making is decided by the government organization to a large extent, and will be promoted by the superior government, their own degree of attention and government levels. Under the Chinese political system, the willingness of grassroots governments to innovate and develop depends to a certain extent on the financial resources and policy support given by higher level governments. Top-down political pressure is an important driver of innovation in public sector governance[23]. Therefore, grassroots social governance innovation cannot be achieved without the support of the higher level government, and the capital investment, policy inclination and resource allocation of the higher level government are the effective support for achieving grassroots social governance innovation[24]. Local governments' self-importance is the internal driving force for promoting innovation in grassroots social governance. Existing studies start with institutional analysis and development framework, and depict the relationship between the government's self-importance and innovation performance in terms of rule supply, policy formulation, policy implementation, and policy supervision[25]. In China's bureaucratic administrative management system, lower-level governments are controlled by higher-level governments, and governments at different levels have different control effectiveness over authoritative resources. Therefore, government levels largely determine the success or failure and effectiveness of grassroots social governance innovation.

**2.3** **Environmental factors and grassroots social governance innovation performance**

Grassroots social governance innovation is inevitably influenced by environmental factors. Since organizations are embedded in the social environment, their basic goal is to adapt, survive and develop their institutional environment to adapt to the changing environment[26]. Based on institutionalism, peer and superior pressure, public pressure, and competitive pressure significantly affect governance performance and innovation level[27]. Some studies have shown that grassroots social governance innovation will be affected by the pressure of inter-governmental competition. In innovation activities, the higher the innovation level of neighboring governments, the greater the pressure on them to promote their own innovation practices, which will lead to the strengthening of innovation practices and improvement of innovation level of governments in the region[28]. In China, local governments respond to public demands in accordance with the requirements of the central government under the pressure of assessment, and the public puts pressure on the government based on efficiently solving the problems of grassroots governance and safeguarding their legitimate rights and interests. The pressure of public demand urges government departments to adopt innovative strategies to improve the performance of grassroots social governance[29]. Open government can be seen as a form of innovation in the public sector, as it requires the government to adopt new technologies with an open attitude[30]. Therefore, open government is an important guarantee for promoting innovation in grassroots social governance.”

We added the following literature:

“20.	Sachan A, Kumar R, Kumar R. Examining the impact of e-government service process on user satisfaction. Journal of Global Operations and Strategic Sourcing. 2018;11(3):321-36.

21.	Lee S, Jung K. The Role of community-led governance in innovation diffusion: The case of RFID Waste pricing system in the Republic of Korea. Sustainability-Basel. 2018;10(9):3125.

22.	Saxena S. Summarizing the decadal literature in open government data (OGD) research: a systematic review. foresight. 2018;20(6):648-64.

23.	Andersen SC, Jakobsen ML. Political Pressure, Conformity Pressure, and Performance Information as Drivers of Public Sector Innovation Adoption. Int Public Manag J. 2018;21(2):213-42. http://doi.org/10.1080/10967494.2018.1425227

24.	Donegan M, Lester TW, Lowe N. Striking a balance: A national assessment of economic development incentives. Urban Aff Rev. 2021;57(3):794-819.

25.	Jin P, Mangla SK, Song M. The power of innovation diffusion: How patent transfer affects urban innovation quality. J Bus Res. 2022;145:414-25.

26.	Adnan HR, Hidayanto AN, Kurnia S. Citizens’ or government’s will? Exploration of why indonesia’s local governments adopt technologies for open government. Sustainability-Basel. 2021;13(20):11197.

27.	Jing Y. Study on the influencing factors of e-government service capacity of provincial governments in China. Front. Edu. Res. 2020;3:12-5.

28.	Manyika J, Lund S, Bughin J. Digital globalization: The new era global flows.: McKinsey Global Institute; 2016.

29.	Cinar E, Trott P, Simms C. A systematic review of barriers to public sector innovation process. Public Manag Rev. 2019;21(2):264-90.

30.	Myeong S, Ahn MJ, Kim Y, Chu S, Suh W. Government Data Performance: The Roles of Technology, Government Capacity, and Globalization through the Effects of National Innovativeness. Sustainability-Basel. 2021;13(22):12589.”

***5.*** ***Please follow the literature review by a clear and concise state of the art analysis. This should clearly show the knowledge gaps identified and link them to your paper goals. Please reason both the novelty and the relevance of your paper goals.***

Answer: Thanks for your suggestion.

First, in the research background of the first paragraph in the introduction, this paper makes a clear and concise analysis of the current situation of grassroots social governance in China, and puts forward the first research goal.

China has made significant efforts to promote grassroots social governance innovation in order to modernize its social governance system and enhance its governance capacity. This has been further advanced by the establishment of awards such as the "China Local Government Innovation Award" and the "China Top Ten Social Governance Innovation Awards". Although governments at all levels have promoted the practice of grassroots social governance innovation, due to the imperfect system and mechanism, imperfect laws and regulations, and blocked participation channels, some grassroots social governance innovation projects have a short-lived phenomenon, and it is difficult to ensure the efficiency and sustainability of innovation projects[4]. Therefore, the first goal that this article addresses is: why do grassroots social governance innovation projects suffer from the same fate of being ineffective despite taking different paths? How to improve the practical efficiency of grassroots social governance innovation projects?

Second，After literature review, this paper studies the current situation of the relevant theories of grassroots social governance innovation performance, and puts forward the second research goal.

Scholars have explored the innovation of grassroots social governance from different perspectives, which is of reference significance to this paper. But there are two limitations to the existing research: First, most researchers only study the innovation performance of grassroots social governance from a single factor, and cannot clearly identify the "interactive relationship" between different factors. This situation often leads to excessive dependence on a single factor in the innovation performance of grassroots social governance, and it is impossible to maximize the efficiency of resource utilization through reasonable allocation of resources. Second, few studies have explored the joint effects of multiple factors, nor have in-depth studies been conducted on the anthems of the differences in grassroots social governance innovation performance, the lack of analysis on the interaction mechanism of multiple complex conditions from the perspective of configuration, the configuration law of conditions is not clear, and the differentiation path and combination of improving grassroots social governance innovation performance need to be studied. Therefore, the second goal that this article addresses is whether and to what extent multiple factors are necessary conditions for achieving high-performance grassroots social governance innovation. How do multiple factors couple and interact to enhance grassroots social governance innovation performance?

6. ***Clearly discuss what the previous studies that you are referring to. What are the Research Gaps/Contributions? Please note that the paper may not be considered further without a clear research gap and novelty of the study.***

Answer: Thanks for your suggestion. We have reviewed the literature and linked the literature with our research to highlight the value contribution and novelty of this research. The specific contents are as follows:

“The academic circles mainly study the innovation of grassroots social governance from three perspectives. The first is to study the barriers to innovation in grassroots social governance from the perspective of problem interpretation. From the government level, the government is the leader of grassroots social governance innovation, and grassroots social governance innovation has a strong political nature, and the government pays more attention to efficiency but ignores democracy[5].It makes the grassroots social governance innovation lack vitality. At the same time, the dislocation and absence of functions of grass-roots governments, insufficient supply of resources, and lagging management reduce the innovation performance to some extent[6].From the perspective of the public, grassroots social governance innovation is easy to be covered up by technocracy and exclusivity, and the public is rarely the core of previous deliberation, decision-making and development[7], and there is no institutional guarantee for public participation in grassroots social governance innovation; From the perspective of innovation itself, grassroots social governance innovation is not sustainable, innovation subjects lack enthusiasm, innovation projects lack operability, and the use of innovative technologies is insufficient. The second is to study the influencing factors of grassroots social governance innovation from the perspective of causality. Based on the political dimension, some scholars have discussed the impact of government authority, political support[8], organizational structure, power structure, legal structure, action structure, grassroots social governance system, and the "trinity" model on grassroots social governance innovation. Other scholars have studied the interactions between personal attributes, political leaders and their environment[9], top-down political mandates and policy pressures, civil rights and civic participation[10], and the overlap of innovative activities with existing social structures and social needs[11]. The net effect of other factors on grassroots social governance innovation. The third is to study the innovation path of grassroots social governance based on the goal-oriented perspective. From the government level, the government should optimize the grid management system in governance innovation, build the network structure of actors, and form innovation synergy[12].The government should further integrate various resources, respect the interests of multiple subjects, strengthen political commitment, optimize the collaborative governance model, improve the grassroots governance innovation system, and form a grassroots governance innovation community. From the public level, improve the enthusiasm of the public to participate in grassroots social governance innovation, improve the "government-society" cooperation mechanism, strengthen technological innovation and application, and stimulate the vitality of grassroots social governance innovation[13];From the perspective of the innovation process, the innovation process of grassroots social governance itself is conducive to fostering a more general democracy, which requires strengthening the use of technology, coordinating the interests of all parties, and improving infrastructure[14].The innovation process needs to be more open to public scrutiny and wider participation.”

We added the following literature:

5.	Stirling A. Towards Innovation Democracy? Participation, Responsibility and Precaution in Innovation Governance. Participation, Responsibility and Precaution in Innovation Governance.(November 2014). SWPS. 2014;24.

6.	Li A. Analysis of the Problems and Countermeasures of Grass-Roots Government in Community Governance. Academic Journal of Management and Social Sciences. 2023;5(1):131-7.

7.	Kearnes M, Chilvers J. Remaking Participation in Science and Democracy. Science, Technology, and Human Values. 2020;45(3).

8.	Cinar E, Trott P, Simms C. A systematic review of barriers to public sector innovation process. Public Manag Rev. 2019;21(2):264-90.

9.	Wynen J, Verhoest K, Ongaro E, Van Thiel S, In CWTC. Innovation-oriented culture in the public sector: Do managerial autonomy and result control lead to innovation? Public Manag Rev. 2014;16(1):45-66.

10.	Crosby BC, T Hart P, Torfing J. Public value creation through collaborative innovation. Public Manag Rev. 2017;19(5):655-69.

11.	Korac S, Saliterer I, Walker RM. Analysing the environmental antecedents of innovation adoption among politicians and public managers. Public Manag Rev. 2017;19(4):566-87.

12.	Mittelstaedt JC. The grid management system in contemporary China: Grass-roots governance in social surveillance and service provision. China Inform. 2022;36(1):3-22.

13.	Huang X, Zhou L. “Paired competition”: A new mechanism for the innovation of urban grassroots governance. Chinese Journal of Sociology. 2023;9(1):3-45.

14.	Smith A, Stirling A. Innovation, sustainability and democracy: An analysis of grassroots contributions. Journal of Self-Governance and Management Economics. 2018;6(1):64-97.

Explanatory note：

Based on this, the main contributions of this paper are as follows: First of all, from the perspective of configuration, this paper discusses the impact of coordination and integration of multiple factors on the innovation performance of grassroots social governance, expands the study based on single variable "net effect" to the study of multi-factor comprehensive effect based on the perspective of configuration, and clarifies the impact of each condition on the innovation performance of grassroots social governance and the linkage interaction between conditions. Secondly, the TOE framework is introduced in this paper to explore the influencing factors of grassroots social governance innovation performance from three aspects: technology, organization and environment, which is conducive to building a multidimensional comprehensive governance model of grassroots social governance innovation. Finally, through configuration analysis, this paper explores the anthegen configuration of the high innovation performance path of grassroots social governance, reveals its core influencing factors and the substitution effect between different paths, proves that there is not only one mode to improve grassroots social governance innovation performance, and provides new ideas for managers to flexibly choose innovation modes and improve grassroots social governance innovation performance.

***6.*** ***There is no flow in the text. It partly depends on the lack of proofreading but also on the fact that many statements and claims are made without being followed up by a clear and logical discussion. It is especially problematic in the Introduction that brings up a number of findings from different areas without linking them together.***

Answer: Thanks for your suggestion. In this paper, the introduction is reorganized and written. Some statements and claims are clearly discussed and linked together. And other parts of the content to sort out and clear ideas. Details are as follows:

“The academic circles mainly study the innovation of grassroots social governance from three perspectives.

The first is to study the barriers to innovation in grassroots social governance from the perspective of problem interpretation. From the government level, the government is the leader of grassroots social governance innovation, and grassroots social governance innovation has a strong political nature, and the government pays more attention to efficiency but ignores democracy[5].It makes the grassroots social governance innovation lack vitality. At the same time, the dislocation and absence of functions of grass-roots governments, insufficient supply of resources, and lagging management reduce the innovation performance to some extent[6].From the perspective of the public, grassroots social governance innovation is easy to be covered up by technocracy and exclusivity, and the public is rarely the core of previous deliberation, decision-making and development[7], and there is no institutional guarantee for public participation in grassroots social governance innovation; From the perspective of innovation itself, grassroots social governance innovation is not sustainable, innovation subjects lack enthusiasm, innovation projects lack operability, and the use of innovative technologies is insufficient. The second is to study the influencing factors of grassroots social governance innovation from the perspective of causality. Based on the political dimension, some scholars have discussed the impact of government authority, political support[8], organizational structure, power structure, legal structure, action structure, grassroots social governance system, and the "trinity" model on grassroots social governance innovation. Other scholars have studied the interactions between personal attributes, political leaders and their environment[9], top-down political mandates and policy pressures, civil rights and civic participation[10], and the overlap of innovative activities with existing social structures and social needs[11]. The net effect of other factors on grassroots social governance innovation. The third is to study the innovation path of grassroots social governance based on the goal-oriented perspective. From the government level, the government should optimize the grid management system in governance innovation, build the network structure of actors, and form innovation synergy[12].The government should further integrate various resources, respect the interests of multiple subjects, strengthen political commitment, optimize the collaborative governance model, improve the grassroots governance innovation system, and form a grassroots governance innovation community. From the public level, improve the enthusiasm of the public to participate in grassroots social governance innovation, improve the "government-society" cooperation mechanism, strengthen technological innovation and application, and stimulate the vitality of grassroots social governance innovation[13];From the perspective of the innovation process, the innovation process of grassroots social governance itself is conducive to fostering a more general democracy, which requires strengthening the use of technology, coordinating the interests of all parties, and improving infrastructure[14].The innovation process needs to be more open to public scrutiny and wider participation.”

We added the following literature:

5.	Stirling A. Towards Innovation Democracy? Participation, Responsibility and Precaution in Innovation Governance. Participation, Responsibility and Precaution in Innovation Governance.(November 2014). SWPS. 2014;24.

6.	Li A. Analysis of the Problems and Countermeasures of Grass-Roots Government in Community Governance. Academic Journal of Management and Social Sciences. 2023;5(1):131-7.

7.	Kearnes M, Chilvers J. Remaking Participation in Science and Democracy. Science, Technology, and Human Values. 2020;45(3).

8.	Cinar E, Trott P, Simms C. A systematic review of barriers to public sector innovation process. Public Manag Rev. 2019;21(2):264-90.

9.	Wynen J, Verhoest K, Ongaro E, Van Thiel S, In CWTC. Innovation-oriented culture in the public sector: Do managerial autonomy and result control lead to innovation? Public Manag Rev. 2014;16(1):45-66.

10.	Crosby BC, T Hart P, Torfing J. Public value creation through collaborative innovation. Public Manag Rev. 2017;19(5):655-69.

11.	Korac S, Saliterer I, Walker RM. Analysing the environmental antecedents of innovation adoption among politicians and public managers. Public Manag Rev. 2017;19(4):566-87.

12.	Mittelstaedt JC. The grid management system in contemporary China: Grass-roots governance in social surveillance and service provision. China Inform. 2022;36(1):3-22.

13.	Huang X, Zhou L. “Paired competition”: A new mechanism for the innovation of urban grassroots governance. Chinese Journal of Sociology. 2023;9(1):3-45.

14.	Smith A, Stirling A. Innovation, sustainability and democracy: An analysis of grassroots contributions. Journal of Self-Governance and Management Economics. 2018;6(1):64-97.
